# Comparison of High-Resolution Computed Tomography and Real-Time Reverse Transcriptase Polymerase Chain Reaction in Diagnosis of COVID-19 Pneumonia in Intensive Care Unit Population

**DOI:** 10.7759/cureus.13602

**Published:** 2021-02-28

**Authors:** Hafiz G Murtaza, Nasir Javed, Ahtesham Iqbal, Moazma Ramzan, Omair ul haq Lodhi, Sadaf Majid, Kiran Abbas, Abdul Rehman

**Affiliations:** 1 Critical Care Medicine, Shifa International Hospital Islamabad, Islamabad, PAK; 2 Internal Medicine, Shifa International Hospital Islamabad, Islamabad, PAK; 3 Neurology, Shifa International Hospital Islamabad, Islamabad, PAK; 4 Medicine, Jinnah Postgraduate Medical Centre, Karachi, PAK

**Keywords:** polymerase chain reactions, diagnostic imaging, computed tomography, x-ray

## Abstract

Introduction

The efficacy of high-resolution computed tomography (HRCT) chest in common respiratory infections is well-established; however, its use in the diagnosis of COVID-19 pneumonia is less popular. The previous studies have failed to establish the efficacy of HRCT in the diagnosis of COVID-19 pneumonia.

Objective

The current study aimed to assess the efficacy of HRCT as compared to a polymerase chain reaction (PCR) in diagnosing COVID-19 pneumonia in patients in our setting.

Methodology

A prospective observational study was conducted at the Department of Chest Medicine, Shifa International Hospital from April 2020 to December 2020. A total of 250 patients were admitted to medical intensive care units. Findings of HRCT and PCR were documented. The accuracy of HRCT compared with PCR was assessed. Data were analyzed using SPSS version 24 (IBM Corp., Armonk, NY).

Results

COVID-19 infection was more prevalent in male patients (62.8% vs 37.2%). The mean age was 60 years (interquartile range, IQR, 49-72). Sensitivity and specificity of HRCT segregated into typical, indeterminate, and atypical HRCT were (94.8%, 56.8%), (92.7%, 47.2%), and (91.7%, 76.8%), respectively. The positive predictive value for typical HRCT was 84.3% (p≤0.001).

Conclusion

We concluded that typical HRCT findings have diagnostic utility in the diagnosis of COVID pneumonia. Similarly, a negative HRCT chest reliably excludes the possibility of COVID pneumonia. HRCT chest is a reliable alternative to RT-PCR.

## Introduction

By the end of 2019, a unique viral infection started to spread all across the globe which was initially reported in Wuhan, China [1]. It was suspected to have spread from phinolphus bats to humans and was regarded as SARS-CoV-2 or COVID-19 infection [2]. As of now, COVID-19 has infected 89.7 million people all around the world with 1.93 million deaths. In Pakistan, 502,000 cases have been reported with 10,644 deaths, nationally [3,4].

The COVID-19 infection is mainly spread from human to human via respiratory droplets during sneezing and coughing. The average incubation period of this virus is between 2.1 days and 11.1 days during which the infected person remains asymptomatic. This greatly contributes to the transmission of the virus [5].

Currently, research is focused on developing an effective vaccine against the virus, however, the results have been inconsistent. Therefore, it is of extreme importance to detect the infected patients and minimize their exposure to the healthy population. Immediate isolation of patients infected with the COVID-19 virus is the most effective way to stop the spread to a further population [6,7].

Currently, the COVID-19 infection is diagnosed with the aid of detection of viral nucleic acids using real-time polymerase chain reaction (RT-PCR) or gene sequencing for respiratory or blood specimens [5]. However, there are some challenges associated with these diagnostic modalities. For instance, sample collection and transportation is restricted by limited workforce and unavailability of PCR kit. The total positive rate of RT-PCR for COVID-19 throat swab cultures is reported to be somewhere between 30% and 60% [6].

On the other hand, a recent study by Qureshi et al. reported that high-resolution computed tomography (HRCT) thorax is a substantially useful modality in establishing the diagnosis of COVID-19 pneumonia [8]. Furthermore, HRCT is less time-consuming and may reveal abnormalities in the lung parenchyma consistent with features of COVID-19 pneumonia in individuals with negative PCR of nasal or throat swab culture results [7]. The comparative studies between HRCT chest and RT-PCR in the ICU population have not been carried out so far. That is why we are conducting this study to analyze the diagnostic value of HRCT chest as compared to RT-PCR in the ICU population and also to determine the diagnostic utility of HRCT chest in comparison to RT-PCR in the diagnosis of COVID pneumonia in the ICU population.

## Materials and methods

An observational prospective research was undertaken at the Intensive care units, Shifa International Hospital, Islamabad, Pakistan between April 2020 to December 2020 for a duration of nine months. The study was first approved by the ethical committee of Shifa International hospital with IRB reference number 128-948-2020. Informed verbal consent was taken from all patients. The study included all patients presenting with signs and symptoms of COVID-19 infection including cough, shortness of breath, fever, and chest pain, admitted to the Intensive care units of the hospital. A non-probability consecutive sampling technique was applied.

Sociodemographic variables and clinical history of patients were obtained from the guardians in detail and recorded in a predefined proforma. The variables recorded included, gender, age, marital status, presenting complaints, duration of symptoms, and comorbidities. Only patients with HRCT chest and RT-PCR test on nasopharyngeal swab findings of no more than two days old since the admission to ICU were included in the study. The sample size was calculated using an electronic calculator. A total of 250 patients fulfilled the eligibility criteria for participation in the study.

A patient was considered as a “confirmed” case of COVID-19 pneumonia if a single positive RT-PCR result was positive. A patient with two consecutive negative RT-PCR results two days apart were considered as “negative” cases. Patients were tested for COVID-19 pneumonia using real-time reverse transcriptase-polymerase chain reaction (RT-PCR) tests to detect viral nucleic acid and HRCT Chest.

All HRCT chests were performed in the radiology department by an experienced radiologist. Patients were asked to lay in the supine position with a tube voltage of 120 kV and slice thickness of 1 cm [8].

HRCT findings were categorized into four groups, i.e., (1) typical, (2) indeterminate, (3) atypical, and (4) negative for COVID-19. The main findings of HRCT in patients with COVID-19 pneumonia were defined as the presence of bilateral peripheral multilobar or multifocal ground-glass opacities (GGO) with or without consolidation with interseptal thickening (IST) or peripheral bilateral GGO with or without consolidation with IST or typical crazy paving pattern or reverse halo sign or other findings of organizing pneumonia.

HRCT intermediate for COVID-19 was defined as the presence of bilateral GGO and consolidation or multi-focal, perihilar, or unilateral GGO with or without peripheral consolidation without any particular distribution or a few very small non-rounded or non-peripheral GGO. An atypical COVID-19 picture was characterized by consolidation in the isolated lobe without any GGO, small discrete nodules, cavitation, thickening of the septa, or lung effusion.

HRCT chest was taken as an index test because, in comparison to RRT-PCR and immunological testing, it is non-invasive, readily available, easy to perform, replicable, and does not depend on the duration of illness. Its reported sensitivity and specificity are 91.9% and 25.1%, respectively [8].

Data analysis was done by using statistical software for social sciences (SPSS version 24, IBM Corp., Armonk, NY). We presented categorical data in frequencies and percentages and age in median and interquartile range (IQR) being non-normal distribution. Considering the RT-PCR test as a reference, we calculated sensitivity, specificity, positive predictive values using contingency tables with 95% confidence intervals. Statistical significance was considered at a p-value of <0.05.

## Results

**Figure 1:**
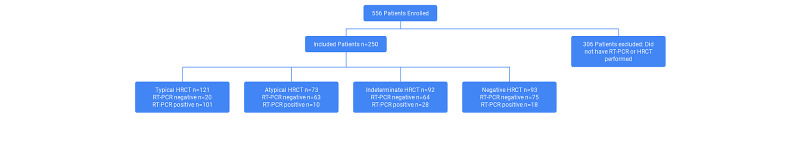
Flow chart of study participants HRCT: high-resolution computed tomography, RT-PCR: real-time polymerase chain reaction.

Most of our study population consisted of males 157 (62.8%) as compared to females 93 (37.2%). The mean age was 60 years (IQR 49-72). We divided our population into two age groups; ≥60 years and <60 years. Amongst males, most of the patients were ≥60 years (54.8 vs 45.2) whereas in the female group most of the patients were <60 years (51.6 vs 48.4). Negative PCR results were 50.8% in < 60 years patients and 55.6% in ≥60 years age group; however, it was not statistically significant (p=0.375; Figure 1).

This study reported that the sensitivity and specificity of typical and atypical HRCT in comparison with RRT-PCR results were 91% and 76%, respectively. The positive predictive value was 83.4% versus the negative predictive value which was 86.3%, p-value < 0.005 (Table 1). 

**Table 1:** Sensitivity and specificity of typical and atypical HRCT in comparison with RT-PCR outcomes HRCT: high-resolution computed tomography, RT-PCR: real-time polymerase chain reaction.

Test Outcome	PCR Positive	PCR Negative			
HRCT typical	101	20	Positive predictive value=83.4%		
						HRCT atypical	10	63	Negative predictive value=86.3%		
	Sensitivity=91%	Specificity=76%	p-Value < 0.005								

The sensitivity and specificity of typical and indeterminate HRCT in comparison with RRT-PCR results were 78.2% and 76.1%, respectively. The positive predictive was 83.4% versus the negative predictive value which was 69.6%, p-value < 0.005 (Table 2).

**Table 2:** Sensitivity and specificity of typical and Indeterminate HRCT in comparison with RRT-PCR outcomes HRCT: high-resolution computed tomography, RRT-PCR: real-time reverse transcriptase-polymerase chain reaction.

Test Outcome	PCR Positive	PCR Negative			
HRCT typical	101	20	Positive predictive value=83.4%		
						HRCT indeterminate	28	64	Negative predictive value=69.6%		
	Sensitivity=78.2%	Specificity=76.1%	p-Value < 0.005								

The sensitivity and specificity of typical and negative HRCT in comparison with RRT-PCR results were 84.5% and 78.9%, respectively. The positive predictive was 83.4% versus the negative predictive value which was 80.6%, p-value < 0.005 (Table 3).

**Table 3:** Sensitivity and specificity of typical and negative HRCT in comparison with RRT-PCR outcomes HRCT: high-resolution computed tomography, RRT-PCR: real-time reverse transcriptase-polymerase chain reaction.

Test Outcome	PCR Positive	PCR Negative			
HRCT typical	101	20	Positive predictive value=83.4%		
						HRCT negative	18	75	Negative predictive value=80.6%		
	Sensitivity=84.5%	Specificity=78.9%	p-Value < 0.005								

## Discussion

The current study evaluated the prospects of HRCT being used as a diagnostic modality in patients suspected of COVID-19 infection. Due to the reduced sensitivity of RT PCR, many patients remain unidentified. This delays treatment and also contributes to the transmission of COVID-19 infection [8-10]. Computed tomography is technically not as demanding as RRT-PCR. It is routinely used in the diagnosis of pneumonia. It also provides results faster as compared to COVID-19 PCR [11-13].

A typical HRCT picture of COVID-19 pneumonia as reported by recent studies shows GGO with concomitant multifocal patchy consolidates or interstitial or parenchymal changes [7]. Similar findings in HRCT of patients with negative RRT-PCR reports were observed indicating that HRCT can diagnose the abnormalities in the lung of patients infected with COVID-19 even when PCR is false negative [12]. A meta-analysis has concluded the sensitivity of RT-PCR on the nasopharyngeal swab and HRCT chest 68.1% to 78% and 91.9%, respectively, but, the accuracy of RT-PCR depends on the adequacy of sample and duration of illness and takes hours in reporting [9,10].

In our study, males were more commonly affected as compared to females (62.8% vs 37.2%). COVID-19 is common in males as they have more expression of receptors for coronavirus attachment (ACE 2), higher chances of smoking and alcoholism. Females are more resilient to COVID-19 infection and responsible behavior to COVID-19 pandemic as compared to males [14]. Typical HRCT findings had been more popular in our study population and had a statistically significant association with PCR results. The sensitivity of typical HRCT was 91.7-94.8% which is comparable to the results of a meta-analysis of 16 studies that showed that the HRCT chest is 86-96% sensitive in the diagnosis of COVID-19 pneumonia. This replication of results is due to the fact that bilateral GGOs and multilobe consolidations were typical findings on the HRCT chest and we adopted the same pattern to define a typical HRCT chest [15]. Salehi et al. have also described the same findings in their systematic review to define typical findings on HRCT [16]. Our results showed quite a higher 56.8% specificity of HRCT chest which is higher than 33% reported by Xu et al. in their meta-analysis [15]. This difference may be due to superimposed viral pneumonia which can reduce specificity of HRCT chest, heterogeneity of expertise of radiologists, and severity of illness in the COVID-19 population.

In a local study by Qureshi et al., HRCT reported sensitivity and specificity of 97.41% and 80%, respectively, with a positive predictive value of 99.12% and a negative predictive value of 57.14%. Furthermore, they reported the diagnostic accuracy of HRCT for COVID-19 to be 96.69% [8].

This concludes that in patients with suspected COVID-19 pneumonia, the HRCT chest in addition to RRT-PCR can be used for the diagnosis of COVID-19 pneumonia. Long et al., reveals that the sensitivity of CT scan was 97.2%, whereas the PCR sensitivity was 83.3%. The study authors recommend that it is better to isolate patients with typical findings of COVID-19 on chest CT even when PCR is negative for COVID-19 nucleic acids [13].

## Conclusions

In conclusion, typical HRCT findings have diagnostic utility in the diagnosis of COVID-19 pneumonia. Similarly, negative HRCT chest reliably excludes the possibility of COVID-19 pneumonia. Although RT-PCR is a gold standard test to diagnose COVID-19, HRCT can be useful in those circumstances where RT-PCR is awaited and isolation of COVID-19 cases is a concern. Sometimes, there is high clinical suspicion of COVID-19 but RT-PCR may be falsely negative. Moreover, resources for RT-PCR may run short in view of the increasing number of cases at some centers. In such circumstances, HRCT findings can be a possible alternative.
